# Using improved CRITIC method to evaluate thermal coal suppliers

**DOI:** 10.1038/s41598-023-27495-6

**Published:** 2023-01-05

**Authors:** Shuheng Zhong, Yiyu Chen, Yinjun Miao

**Affiliations:** School of Energy and Mining Engineering, China University of Mining & Technology(Beijing), Beijing, 100083 China

**Keywords:** Energy infrastructure, Fossil fuels

## Abstract

Nowadays the complex international political situation has caused the shortage of coal supply in the European region. Scholars have done a lot of research on supplier evaluation. However, these studies don’t reflect the variability of the indicators, such as interruption caused by recent war. Coal-electricity-integrated companies have a large demand for thermal coal and high requirements for stable supply, so they need to conduct timely and effective short-term evaluation of suppliers. This paper improves the CRITIC method and uses short-term transaction data for a coal-electricity-integrated firm to evaluate its coal suppliers. The results show that the improved CRITIC method effectively avoids the problem of weight changes caused by conflicting value ranges of indicators, and its evaluation results are more consistent with the actual situation, which can meet the requirements of large coal enterprises for evaluating suppliers.

## Introduction

Vertical integration of coal and electricity is the development trend of China's coal and power industry and is an effective means to alleviate the conflict between coal and electricity^1^. By integrating resources and production command, the enterprise effectively reduces production and internal transaction costs and maximizes residual income and optimizing internal distribution in the chain of integrated operations^2^. Vertical integration of coal and electricity can also enhance the adaptability of coal and electricity companies to market fluctuations and ensure a stable supply of coal in the form of internal transactions, which in turn will guarantee the supply of electricity.

**Figure 1:**
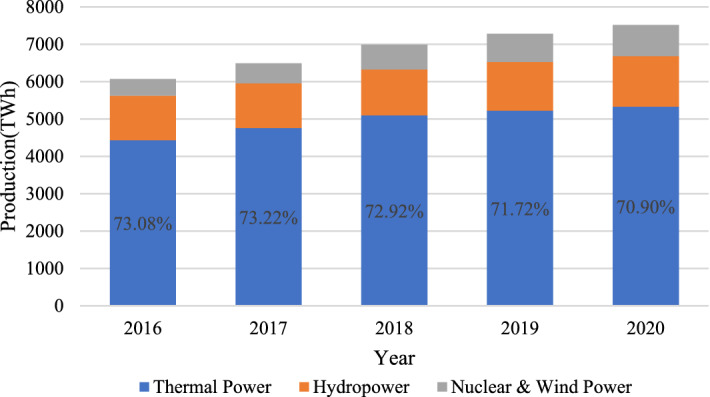
The structure of electricity production in China.

**Figure 2:**
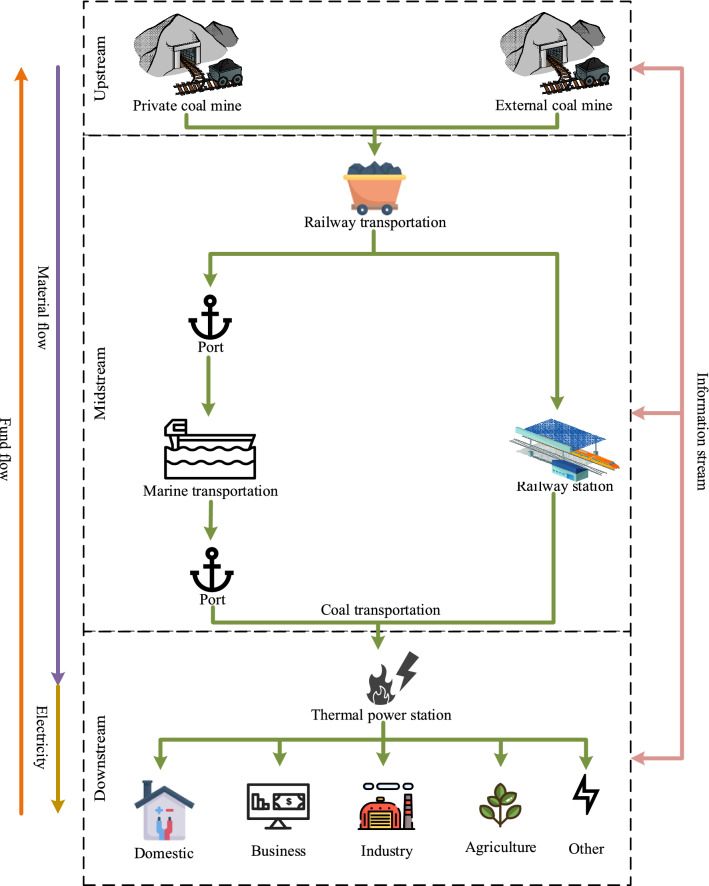
The structure of coal supply chain in coal-electricity-integrated enterprises.

However, China's electricity supply is still dominated by thermal power generation, as shown in Fig. 1. Although the proportion of thermal power in all power production has generally declined between 2016 and 2020, it still accounts for more than 70%. Therefore, China’s power generation industry is still highly dependent on a stable supply of thermal coal. Due to the wide scope of business and large scale of operation, coal-electricity-integrated enterprises usually can’t meet the coal demand by relying only on the supply of their own coal mines, so they also need to purchase outsourced coal^3^. Its coal supply structure is shown in Fig. 2. Due to the market-based pricing mechanism of commodity coal, its price is prone to fluctuations due to external factors^4^. Especially when the price of commodity coal rises, much higher than the long-term contract price, the outsourcing coal suppliers may reduce their shipments to the coal-electricity-integrated enterprises after measuring their default costs and benefits, resulting in shortage of coal supply and affecting the companys’ production and operation, at which time the coal-electricity-integrated enterprises will encounter a difficult period of coal procurement. When the difficult period is over, in order to optimize their coal supply chain, the enterprises will prioritize the allocation of orders to high-quality suppliers based on the shipments data during the difficult period.

The evaluation of coal suppliers in coal-electricity-integrated company is studied in this paper for the following reasons:

1. Due to the fluctuation of coal price, the vertically integrated enterprises will encounter the difficult period of coal procurement, i.e. the outsourcing coal suppliers will choose to breach the contract for their own interest when the market price of coal is higher than the contract price and will not complete the order.
2. Purchased coal accounts for a portion of the coal used by a vertically integrated coal and electricity enterprise. And the order fulfillment of the outsourced coal suppliers affects the normal operation of this enterprise.
3. The vertically coal-electricity-integrated companies, as the demand side of coal, need to assign orders to superior suppliers on a priority basis based on past supplier performance.
4. The vertically integrated coal and electricity enterprises are usually large in scale and have a high degree of information technology platform development. The performance data of its coal suppliers is stored in the platform's back-end database. The evaluation model is required to update and display the evaluation results based on the changes of the platform background data, so a auxiliary decision-making module can be established in the information technology platform.

The purpose and main task of this paper is to seek a reasonable and accurate short-term supplier evaluation method that meets the requirements for evaluating the performance of thermal coal suppliers. The evaluation results can be used as the basis for coal-electricity integration enterprises to allocate coal orders. The method must be suitable for dynamic and objective evaluation data and meets the requirements of the company’s supply chain management system (SCM) for real-time update and display of evaluation results.

The CRITIC method is a comprehensive and objective weighting method. Compared with the entropy weight method and the FANMA method, it not only considers the dispersion of the data of each criterion evaluation set, but also considers the correlation of the criteria, and the calculation method is simple. When using CRITIC method to evaluate thermal coal suppliers, it is found that the conflicting value range of the indicators will lead to the problem of inaccurate calculation of the amount of information in the criteria, but the existing research literature rarely mentions this phenomenon. Therefore, this paper proposes an improved CRITIC method to evaluate and select suppliers based on the short-term delivery data of coal-electricity integrated enterprises. In the improved CRITIC method, the conflicting value ranges of indicators ignored in the traditional CRITIC method is considered. By using improved CRITIC method, the problem of inaccurate calculation of information content was solved.

## Literature review

### Coal-electricity integration studies

The mismatch of institutional change between the coal industry and the thermal power industry has led to a conflicting relationship between the two industries. Scholars have mainly studied the vertical integration of coal and electricity as a method to solve the conflict relationship between coal and electricity. Linmei Yuan^5^ analyzed the coal power industry through the SCP model, and believed that the vertical integration of coal power is an effective way to alleviate the contradiction between coal and electricity. Shukui Yu^6^ analyzed the significance, existing problems and development countermeasures of coal enterprises implementing vertical integration strategy. From the perspective of bilateral matching of coal and electricity, Rui Nie^7^ constructed a transaction model of coal and electricity enterprises, designed a price generation mechanism for thermal coal, and put forward countermeasures to ensure the smooth operation of the thermal coal market. Daqing Zhu^8^ argued that the current level of coal-electricity integration in China is still low, and accelerating the process of integration requires enhancing the short-term market power of power coal and coal-fired power producers.

As for the advantages of vertical integration of coal and electricity, Hongji Shi^9^ established a game model of coal-electricity integration and concluded that integration can improve corporate profits. Yujia Wang^10^ found that vertical integration of coal and electricity can optimize resource allocation and improvethe productivity of enterprises. David Brown^11^ found that vertical integration of electricity sector reduced retail electricity prices while increasing industry capacity investment, wholesale electricity supply and consumer surplus. Hongye Guo^12^ used the MILP model to simulate the integrated market equilibrium. Through case research, it is found that the introduction of vertical integration in the power industry can achieve Pareto optimality and alleviate the abuse of market power.

Scholars have also analyzed the problems that can result from the vertical integration of coal and electricity. Hongyi Li^13^ believed that compared with developed countries, vertical integration in developing countries improves the ability of insiders to obtain private benefits and has negatively effects on the efficiency of enterprises. IN Pinopoulos^14^ studied the welfare effect of the integration of upstream and downstream suppliers through linear tariffs, and found that when downstream enterprises have higher bargaining power, vertical integration will reduce the welfare effect. From the perspective of risk, Jinfang Yao^15^ analyzed the relationship between the degree of enterprise integration and financial risk through a multiple linear regression model and concluded that the higher the degree of vertical integration of coal enterprises, the greater the financial risk faced by the enterprises.

### Studies on supplier evaluation and selection

The supplier evaluation and selection problem is often viewed as a multi-criteria decision problem (MCDM). Although scholars have already done relatively mature research on supplier evaluation, they mainly consider long-term factors, but there is not much research on the evaluation of suppliers based on a small amount of data in the short term. The large and comprehensive criteria system in long-term evaluation usually involves much data collection work, such as issuing questionnaires and consulting relevant materials, so it is impossible to evaluate based on data that changes in a short period. For thermal power plant companies, the behavior of their coal suppliers may change with coal price fluctuations in the short term, which is mainly reflected in the coal shipments of suppliers. In addition, with the improvement of the level of informatization, enterprises have also put forward higher requirements for the real-time display and update of the evaluation results. In conclusion, the evaluation based on short-term delivery data of each supplier can meet the needs for optimizing the thermal coal supply chain.

Scholars usually consider factors such as quality, price, and delivery when evaluating suppliers, and there are different criteria according to different evaluation objects, as shown in Table 1. Gamiy^21^ considered coal supply interruption from the perspective of coal mining and self-heating.

**Table 1:** Criteria involved in long term evaluation.

References	Research contents	Considerations	Index properties
Hui Liu, Zhitao Xu, Miao Li^16^	In view of the complexity of gas turbine suppliers, evaluation indicators such as quality, cost, delivery, technology and cooperative services are constructed. The weights of indicators at all levels are determined through ANP, and the dimensional differences of indicators are eliminated with the help of TOPSIS. Finally, the feasibility of the method is verified through example analysis	Pass rate, price level, cost control, on-time delivery rate, order fill rate, R&D cycle time, number of patents, question corresponding time	Quantitative
		Design reliability, quality problem handling, deflating price level, procurement cost level, order flexibility, informatization level, device status, after-sales service level, enthusiasm for meeting, information sharing level	Qualitative
Yongzheng Zhang, Chunming Ye, Xiuli Geng^17^	The generalized Choquet integral of the hesitant fuzzy measure is used to analyze the mutual influence relationship between the indicators and calculate the weight of the indicators. Finally, the improved TODIM method is used to analyze the decision makers’ psychological preference and sort the candidate suppliers. The evaluation model takes into account the decision maker's risk preference, which is ignored in traditional supplier evaluation methods	Pass rate, price level, transport costs, payment term, order flexibility, on-time delivery rate, order completion rate, risk management capability, problem response time, problem solving time, per capita training time, per capita training cost, net sales profit rate, asset-liability ratio, total asset weekly accuracy ratio, revenue growth rate	Quantitative
		Quality stability, price stability, order flexibility, informatization level, supplier reputation, supply qualification compliance	Qualitative
Xin Weng, Wei Dang, Xin Tian^18^	For the actual needs of the manned space station project, Monte Carlo simulation is applied to introduce the uncertainty of supplier behavior. By adopting the game cross-efficiency method, the non-cooperative relationship between suppliers is considered while realizing the mutual evaluation mechanism, and the analysis of the average efficiency value of suppliers is transformed into the analysis of their efficiency distribution	Product qualification rate, price level, on-time delivery rate, R&D investment, problem response time, problem solving time, per capita training time, per capita training cost, net sales rate, asset-liability ratio, total asset turnover rate, revenue growth rate	Quantitative
		Quality stability, price stability, order flexibility, informatization level, supply qualification compliance	Qualitative
Luhua Fan^19^	Suppliers are prioritized through the TOPSIS model based on entropy weight, and suitable suppliers are selected for the enterprise. Finally, using the matrix analysis method, the construction materials are divided into four categories, and the differentiated management of suppliers based on material classification is proposed	Product qualification rate, price level, payment term, on-time delivery rate, geographic location, R&D investment, new product R&D capability, problem response time, problem solving time, channel dependence, transaction frequency, quality certification	Quantitative
		Order flexibility, after-sales service level, historical cooperation time, supplier reputation, service personnel quality and attitude, supplier management and organizational capabilities	Qualitative
Haoyuan Wu, Jian Wang, Rong Li^20^	The TOPSIS method is used to evaluate and rank suppliers, and build an integrated configuration model. On the basis of the configuration model, a multi-objective configuration optimization model of products selected by integrated suppliers is constructed, aiming at the optimal comprehensive capability and cost of the supplier, and the multi-objective optimization model is solved by the NSGA-II algorithm	Product qualification rate, on-time delivery rate, R&D investment, new product R&D capability, employee health and safety, net sales margin, asset-liability ratio, resource consumption, product recycling	Quantitative
		Order flexibility, after-sales service level, employee rights and interests, pollutant discharge, green design	Qualitative

### Methodology for determining indicator weights

In multi-criteria decision problems, it is often necessary to assign different weights to different criteria for the following two purposes. 1. For different research areas and evaluation purposes, different weights are usually assigned to each criterion due to different focus and emphasis. For example, for the evaluation of high-end manufacturing suppliers such as gas turbines, the highest weight is given to technology^16^. However, the evaluation of suppliers in industries that require environmental protection measures, such as papermaking enterprises, will give a higher weight to environmental factors^22^. 2. Making full use of the information in the evaluation data can make the evaluation results more distinguishable. As the evaluation data usually contains some mathematical features, such as variance, extreme deviation, information entropy, etc. Taking information entropy as an example, the greater the information entropy of the criterion the higher its data dispersion, which should be given a higher weight. Commonly used weighting methods include subjective methods, objective methods, and methods in which subjective and objective weights are combined. Representative subjective weighting methods include AHP^23^, DEMATEL^24^, etc. The subjective method can reflect the knowledge and experience of decision-makers, but it is highly subjective. Objective weighting methods, such as the entropy weight method^25^, CRITIC method^26^, etc., can reflect the influence of historical data on the weight of evaluation criteria and are highly objective, but highly dependent on data. The combined method^27^ refers to the combination of subjective and objective weight through additive synthesis, multiplicative synthesis, subjective correction based on objective, range maximization, or other combination methods, is a more holistic approach, but it's a lot of work.

Many scholars use subjective methods to determine weights. Dragan Pamucar^28^ thought that the traditional BWM method ignores the possibility of multiple evaluation criteria with the same meaning in the expert preference. He improved the traditional BWM method, reduced the number of comparisons, and verified the practicability of the improved method through examples. Based on the LBWA method, Sanjib Biswas^29^ analyzed the site selection of colleges and universities from the perspective of business school candidates, and considered that transportation convenience and commuting time are the primary factors. Alptekin Ulutas^30^ used the FUCOM method to evaluate the location of textile manufacturing facilities in a province of Turkey. Compared with the AHP and BWM methods, the FUCOM method has more reliable standard weight coefficients, which reduces the subjective influence and inconsistency of the expert preference on the final value of the standard weight. At the same time, the number of pairwise comparisons of the FUCOM method is significantly reduced.

When it comes to objective weighting methods, C Bai et al.^31^ evaluated the social sustainability of suppliers of an Iranian manufacturing company based on the TODIM method. The TODIM method is based on the value function of prospect theory, and establishes the relative superiority function of a solution compared with other solutions according to the psychological behavior of decision makers, and carries out solution selection according to the magnitude of superiority. MA Kaviani et al.^32^ applied gray system theory to supplier evaluation in the oil and gas industry considering the uncertainty faced by decision makers due to lack of experience and information.Chia-nan Wang et al.^33^ developed a flexible and generalizable supplier evaluation and selection model based on the TOPSIS method for supplier evaluation and selection in the Vietnamese apparel industry. Mukhametzyanov^34^ has conducted a comparative analysis of objective methods for determining criteria weights in MCDM and concluded that the soundness of all objective methods for evaluating criteria weights for MCDM tasks is questionable. Navid Zarbakhshnia et al.^35^ evaluated sustainable suppliers in the plastics industry based on the DEA approach in terms of inputs and outputs.

In terms of mixed subjective and objective evaluation methods, Stojanović I et al.^36^ assessed the logistics performance of the GCC countries based on the CRITIC-MABAC hybrid model, and selected the United Arab Emirates as a regional logistics hub that effectively integrates the GCC into the global supply chain system. Abdulaziz Alossta et al.^37^ solved the site selection problem of emergency medical centers based on the AHP-RAFIS method, and considered that the road network is the best construction site compared with other sites. Ibrahim Bad et al.^38^ used the FUCOM-MARCOS method to evaluate the green innovation capability of the Nigerian textile industry, and established a set of green innovation evaluation models that can be applied to other industries. As evaluation problems often involve fuzzy decision making, fuzzy theory is also widely used, usually in combination with other methods such as Fuzzy-AHP^39^, Fuzzy-TOPSIS^40^ and Fuzzy-TODIM^41^.

In summary, subjective, objective, and combination of subjective and objective weighting methods have their own advantages and disadvantages. Both subjective and subjective–objective-combined weighting methods involve questionnaires, expert consultation and other work, which usually take a long time and a large workload. So they are not suitable for rapid short-term evaluation. In this paper, since the supplier evaluation relied on short-term dynamic objective data, objective weighting method was adopted.

## CRITIC method and improvement

### CRITIC method

The CRITIC method measures weight according to the dispersion degree of the criterion and the conflict degree between criteria. The dispersion degree refers to the difference between the values of a criterion in each evaluation scheme. The conflict degree reflects the amount of similar information between different criteria. If a criterion has a higher dispersion degree and higher conflict degree, its weight should be larger.

Suppose there are n alternatives to be evaluated and p evaluation criteria to form the original data matrix:

1 $$X=\left(\begin{array}{ccc}{x}_{11}& \cdots & {x}_{1p}\\ \vdots & \ddots & \vdots \\ {x}_{n1}& \cdots & {x}_{np}\end{array}\right),$$

where ${x}_{ij}$ represents the value of alternative i regarding the criterion j.

To eliminate the influence of different dimensions on the evaluation results, it is necessary to perform the dimensionless process on each index. Positive normalization is used for positive volume criteria and negative normalization is used for negative volume criteria. Formula is as follows:

2 $$ \begin{gathered} {\text{For}}\,{\text{positive}}\,{\text{indicators:}}\,\,\,\,x_{ij}^{^{\prime}} = \frac{{x_{ij} - \min \{ x_{ij} \} }}{{\max \{ x_{ij} \} - \min \{ x_{ij} \} }} \hfill \\ {\text{For}}\,{\text{the}}\,{\text{negative}}\,{\text{indicators:}}\,\,\,x_{ij}^{^{\prime\prime}} = \frac{{\max \{ x_{ij} \} - x_{ij} }}{{\max \{ x_{ij} \} - \min \{ x_{ij} \} }}. \hfill \\ \end{gathered} $$

The dispersion degree is expressed in the form of standard deviation and is calculated as follows:

3 $$\left\{\begin{array}{c}{\overline{x} }_{j}=\frac{1}{n}{\sum }_{i=1}^{n}{x}_{ij}\\ {S}_{j}=\sqrt{\frac{{\sum }_{i=1}^{n}{({x}_{ij}-\overline{x })}^{2}}{n-1}}\end{array}\right..$$

The conflict degree is calculated by:

4 $${R}_{j}=\sum_{\begin{array}{c}i=1\\ i\ne j\end{array}}^{p}(1-{r}_{ij}),$$

where ${r}_{ij}$ represents the Pearson correlation coefficient of criteria i and j. The calculation formula is as follows:

5 $${r}_{ij}=\frac{\sum_{i=1}^{n}({x}_{i}-\overline{x })({y}_{i}-\overline{y })}{\sqrt{{\sum }_{i-1}^{n}{({x}_{i}-\overline{x })}^{2}}\sqrt{{\sum }_{i-1}^{n}{({y}_{i}-\overline{y })}^{2}}}.$$

The amount of information is calculated as follows:

6 $${C}_{j}={S}_{j}\times {R}_{j}.$$

The weight of the criterion is:

7 $${\omega }_{j}=\frac{{C}_{j}}{{\sum }_{j=1}^{p}{C}_{j}}.$$

### Problems and improvements

If there are n criteria, the value range of Rj is [0, n−1]. When calculating C_j_, according to formula (4), if R_j_ > 1, C_j_ will be larger than it should be after being multiplied; if R_j_ < 1, C_j_ will be smaller than it should be after multiplied. However, if the amount of information in one criterion is enlarged while another is reduced, the weight will be enlarged or reduced correspondingly, which will lead to unreasonable evaluation results.

The cause of this problem is the value range of R_j_. If the value of R_j_ can be limited to [0, 1], the problem can be solved. The calculation of R_j_ can be improved as follows:

8 $${R}_{j}=\prod_{\begin{array}{c}i=1\\ i\ne j\end{array}}^{p}(1-{r}_{ij}).$$

## Case study

**Figure 3:**
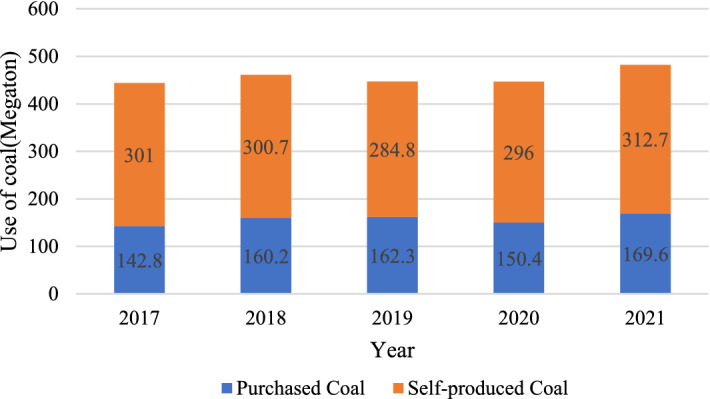
The amount of coal used by a coal-electricity-integrated enterprise in the past 5 years.

**Figure 4:**
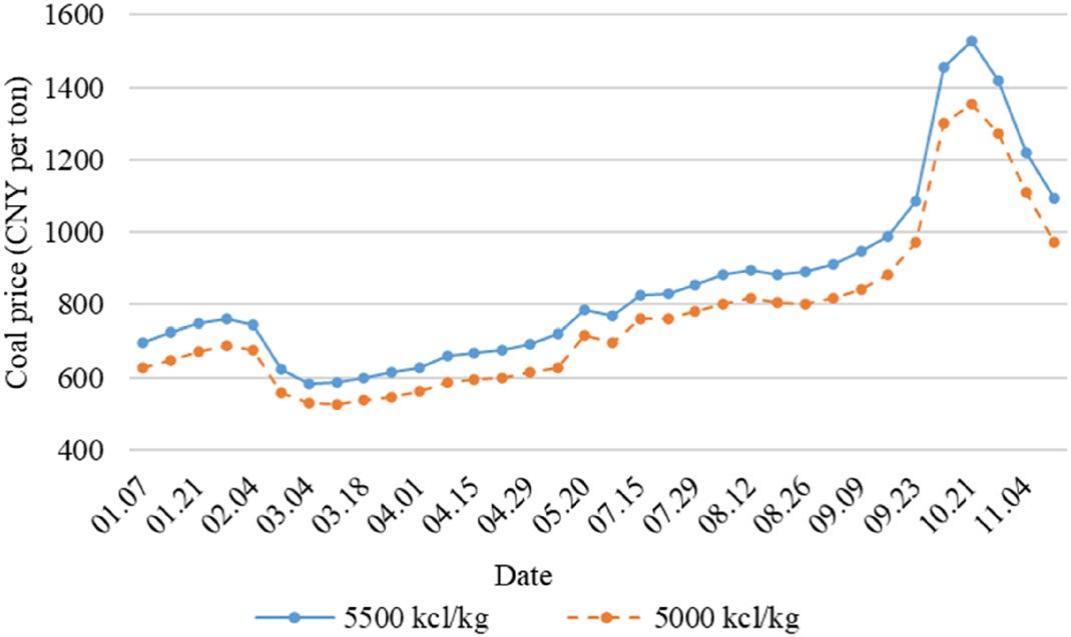
The trend of coal price.

Taking a large integrated coal and power company in China as an example, the amount of self-produced coal and purchased coal in its coal usage over the past 5 years is shown in Fig. 3. Purchased coal still accounts for about 1/3 of the used coal of the coal-electricity-integrated company. Suppliers and the company are usually bounded by long-term contracts for the trading of commodity coal. This coal-electricity-integrated company has 36 suppliers of thermal coal and the price of coal increased successively from September to October 2021 due to various factors such as cold spells, lower coal imports and other reasons, as shown in Fig. 4. As a result of the increase in the price of thermal coal, the coal suppliers reduce their coal shipments to the company for their own benefit, affecting the normal production activities of the company.

The enterprise has a high level of information technology development.Relevant operation data can be obtained directly from the database of the information technology platform. The company wants to establish an auxiliary decision-making module in the platform to realize real-time evaluation, update and display of coal suppliers according to the platform's background data, so as to provide reference for the order allocation work of coal procurement personnel. Each supplier was evaluated using the shipment data during the difficult period of coal acquisition from September to October 2021.The current calculation is the order fulfillment rate of a supplier/total order fulfillment rate of all suppliers. This method does not consider the weight setting, ignores the relative importance between different evaluation criteria. And once the evaluation indexes are increased, the method is no longer applicable.

The improved CRITIC method determines the weights of each indicator based on the variability of indicators and the conflict between indicators, which does not rely on human decision making, is more scientific, and is compatible with the information technology platform. Therefore, in this paper, 36 coal suppliers are evaluated and selected by the improved CRIITC method assigned with the shipment volume of each site in late September, actual shipment volume and planned volume of each site in October as the evaluation data. Due to the small number of indicators and easy availability of quantitative data, a linear weighting method was used to calculate each supplier's score for simplicity, and the calculation process is as follows.

### Raw data processing

Due to the different dimensions of the 3 criteria, normalization is necessary.

$$A={\left[\begin{array}{cccccccccc}0.595& 1.000& 0.380& 0.264& \cdots & 0.000& 0.123& 0.000& 0.000& 0.000\\ 0.901& 0.747& 0.538& 0.208& \cdots & 0.067& 0.067& 0.008& 0.000& 0.000\\ 1.000& 0.691& 0.829& 0.866& \cdots & 0.221& 0.141& 0.036& 0.000& 0.000\end{array}\right]}^{T}$$

where ${a}_{ij}$ represents the normalized value of alternative i regarding the criterion j.

### Weight calculation

The CRITIC method is used to calculate the weight of each evaluation criterion, and the results are shown in Table 2.

**Table 2:** The results of the CRITIC method.

Criterion	Dispersion degree	Conflict degree	Amount of information	Weight
Actual shipment in late September	0.196	0.752	0.147	25.62%
Actual shipment in October	0.241	0.714	0.172	30.00%
Fulfillment ratio of plan in October	0.247	1.033	0.255	44.38%

**Figure 5:**
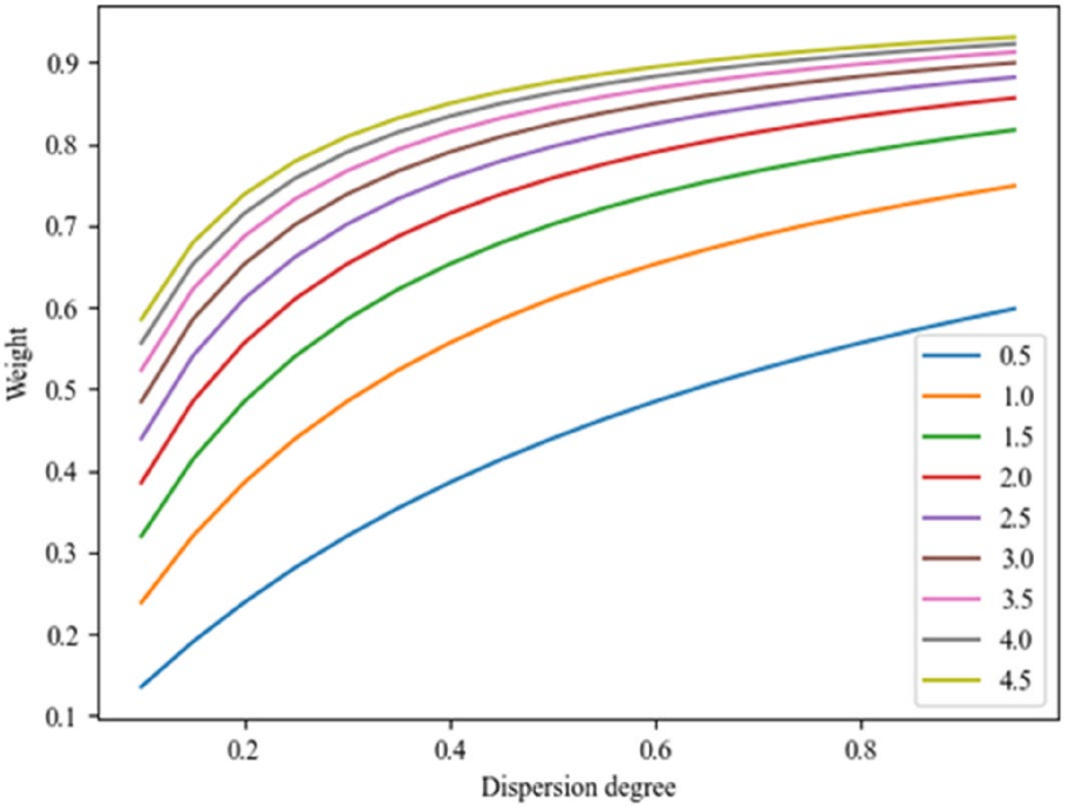
The impact of dispersion degree on weight at different conflict degree levels.

Red indicates a conflicting indicator calculation greater than 1. It can be seen from Table 2 that the conflict degree of the 3rd criterion is larger than 1, which leads to information distortion. As shown in Fig. 5, the slope of the curve changes more when the indicator conflict is at different levels, especially from 0.5 to 2.0, i.e., the influence of the indicator variability on the weights becomes greater. As can be seen in Fig. 5, the variability does not change much in the 0 to 1 interval with the change in the value of conflict. Therefore, the improved CRITIC method was used to calculate the weight of each criterion. The result is shown in Table 3.

**Table 3:** The results of the improved CRITIC method.

Criterion	Dispersion degree	Conflict degree	Amount of information	Weight
Actual shipment in late September	0.196	0.095	0.019	21.03%
Actual shipment in October	0.241	0.083	0.020	22.45%
Fulfillment ratio of plan in October	0.247	0.203	0.050	56.52%

It can be seen from Table 3 that the improved CRITIC method limits the range of conflict values. The correlation analysis shows that the correlation coefficient of dispersion degree and weight is 0.76 and 0.62 in the CRITIC and improved CRITIC method. It can be considered that the improved method reduces the impact of conflict degree on weight.

### Suppliers evaluation based on improved CRITIC method

**Figure 6:**
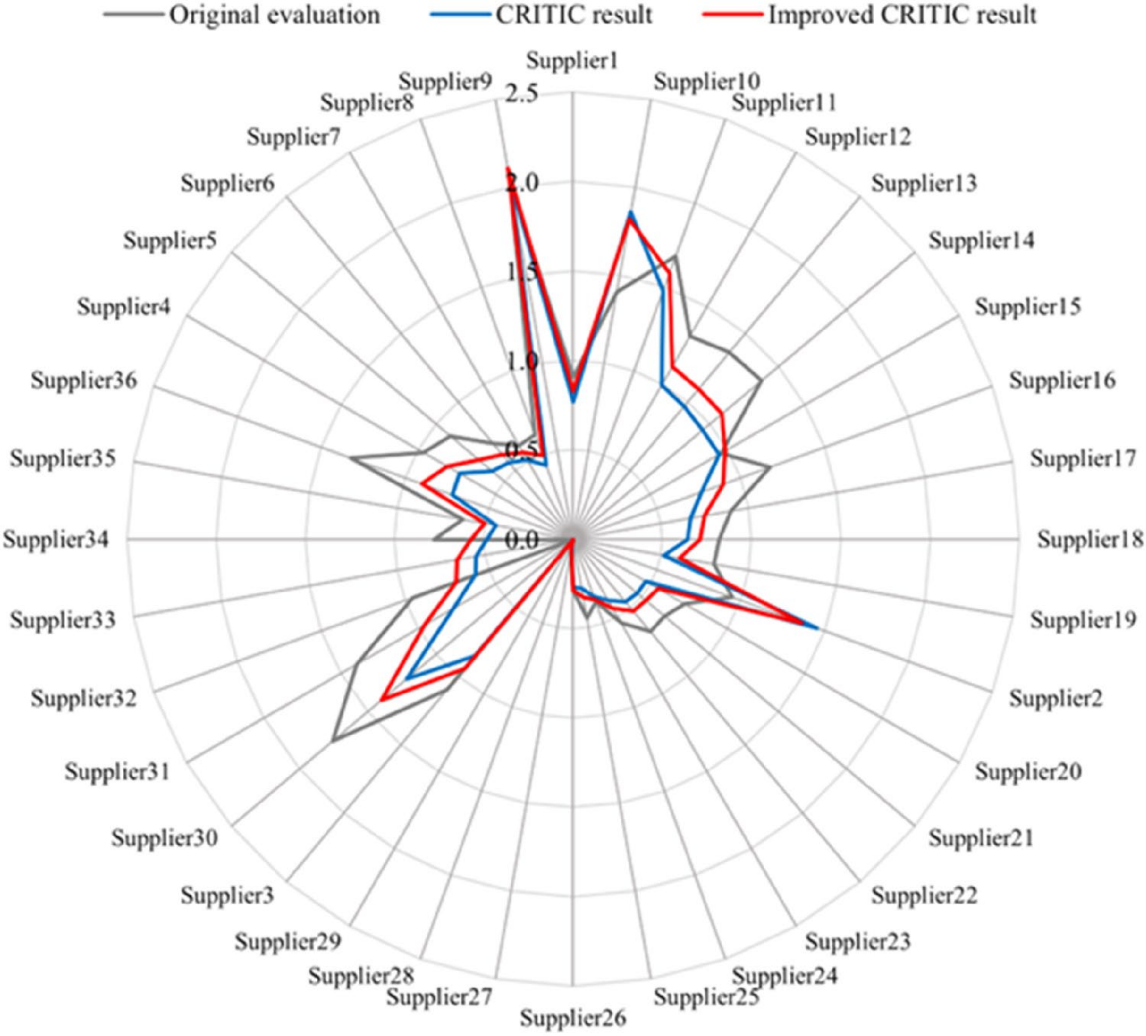
The result of different weighting methods.

The evaluation result based on the weight method of CRITIC and improved CRITIC and the result of the original method is shown in Fig. 6.

Comparing the results of different methods, the ranking changes of each supplier are shown in Table 4. Number in parenthesis is the ranking changes of the supplier. The first number is the ranking change of the evaluation results weighted by the CRITIC method compared to the original results. The second number is the ranking change of results weighted by the CRITIC method compared to the improved CRITIC method.

**Table 4:** The ranking changes of each supplier.

Supplier	Ranking change	Supplier	Ranking change
1	(4, − 2)	19	(− 2, 0)
2	(11, − 1)	20	(− 3, 0)
3	(1, − 1)	21	(− 1, 0)
4	(− 2, − 1)	22	(− 3, 0)
5	(0, − 1)	23	(− 1, 0)
6	(3, − 1)	24	(0, 0)
7	(5, − 1)	25	(− 2, 0)
8	(− 1, − 1)	26	(− 1, 0)
9	(0, 0)	27	(− 1, 0)
10	(2, 0)	28	(0, 0)
11	(0, 0)	29	(0, 0)
12	(3, 0)	30	(− 3, 1)
13	(0, 0)	31	(− 6, 1)
14	(− 2, 0)	32	(− 5, 1)
15	(4, 0)	33	(13, 1)
16	(− 3, 0)	34	(− 3, 1)
17	(1, 0)	35	(− 3, 1)
18	(2, 0)	36	(− 7, 3)

## Analysis and discussion

Comparing the result of the original evaluation method and CRITIC weighting method, it is obvious that suppliers 2 and 33 had a big rise in ranking. Supplier 2 ranked 4/36, 11 places upwards. The reason is that the original method only considered the shipment data in October while neglecting the data in late September. Supplier 33 ranks 21/36, 13 places upwards. Supplier 31 and Supplier 36 have dropped more in the ranking, ranking 11th and 15th in the CRITIC result, 6 and 7 places downwards respectively. The reason is that the suppliers delivered few coals when the company was short of coal in September.

Comparing the result of the CRITIC weighting method and the improved method, suppliers have little change in ranking. In this case, the weight change of the three cri-teria is − 4.59%, − 7.55%, and + 12.14% respectively. As is shown in Table 4, Supplier 36, which rose the most, rose 3 places, while Supplier 1 dropped the most, dropping 2 places.

After the internal discussion of the thermal power company, it is believed that the evaluation results of the improved CRITIC method are the most realistic.

This paper uses the linear weighting method to evaluate and rank suppliers based on evaluation index data, because the case has only three indicators and the linear weighting method is simple to calculate. TOPSIS, a commonly used supplier evaluation method^42^, calculates the relative closeness of each evaluation method to the positive and negative ideal solution to rank each supplier to select the best supplier. However, in the application scenario of this paper, as it is a short-term evaluation, the evaluation results need to be updated frequently based on the constantly changing short-term evaluation data, and TOPSIS may cause the reverse ranking phenomenon when the evaluation data changes frequently. Therefore, if the focus is only put on the selection of the best supplier over time, TOPSIS method can be adopted. Besides the best supplier, if it is also necessary to focus on changes in the ranking of suppliers, other methods need to be considered.

The subject of this paper is a coal-electricity-integrated enterprise, whose supply chain management system currently provides data including two indicators of the planned order quantity and actual shipment quantity of each supplier each month. As late September and October were the difficult period for the enterprise to procure coal, some suppliers refused to fulfil their long-term contracts and reduced their coal supply to the enterprise. The company would like to assess the performance of its suppliers based on data from this difficult period. In order to optimise its coal supply chain, the company would allocate more coal orders to stable suppliers based on the results of the assessment. Taking the results of the improved CRITIC method described above as an example, the highest ranked supplier during the difficult coal procurement period from late September to October is Supplier 9, and the relatively low ranked suppliers are Suppliers 28 and 29. Therefore, when placing coal orders in the following November, the coal-electricity-integrated company will increase the coal order quantity for Supplier 9 and decrease the coal order quantity for Suppliers 28 and 29 in order to establish a more stable coal supply chain.

## Conclusion and outlook

Purchased coal accounts for a certain proportion of coal used by vertically integrated coal and electricity companies. Coal and electricity vertically integrated enterprises carry out thermal coal suppliers evaluation to help optimize their supply chain structure. Weight assignment based on the CRITIC method, which relies solely on the variability and conflict of indicators, excludes the influence of subjective factors. The improved CRITIC method solves the problem of distortion of information quantity caused by conflicting value ranges of indicators in the traditional CRITIC method, and improves the theory of weight calculation. From the empirical analysis, it can be seen that the short-term supplier evaluation based on the improved CRITIC method empowerment is objective and avoids unreasonable results due to subjective bias.

This paper has the following limitaions:

Evaluation indicators and data are few. In the future, with the construction of the company’s SCM platform and the continuous improvement of the system's back-end data, more evaluation indicators can be incorporated into the evaluation system.

Due to the real-time update of the data of the enterprise supply chain management system, the evaluation indicators, the number of evaluation schemes and the data in the evaluation model are in the process of dynamic change. The subjective weighting method, which requires questionnaires or expert scoring, usually takes a long time and cannot meet the needs of SCM to update the evaluation results instantly and dynamically, so the objective weighting method is chosen in this paper to determine the weights. However, a common drawback of the objective weighting method is that if an evaluation indicator is 20–30% higher than other indicators, the weight of that indicator will rise significantly, which is unreasonable. In the future, if the SCM system can integrate functions such as expert scoring into the system to reduce the time and workload required to determine subjective weights, then a combination of subjective and objective weighting methods can be considered in order to obtain more reasonable evaluation results.

The evaluation model is an important part of SCM and enables real-time updating and presentation of the evaluation results based on updates of the back-end data. The evaluation results are recognised by the empirically analysed company and have been used within the company. Therefore, the evaluation method proposed in this paper has a certain degree of practicality. In addition, the main difference between different countries/industries is that the evaluation indicators are different, and as long as the evaluation indicators have quantitative and objective data, the improved CRITIC method can be applied, which makes the method highly scalable. For different countries/industries, the CRITIC method can also be modified by the subjective weighting method to obtain more reasonable weights.

Due to the current general increase in the degree of informatization in various enterprises, the improved CRITIC method proposed in this paper provides some implications for the construction of order allocation decision-making platforms and SCM in other enterprises.

## Data Availability

The data used to support the findings of this study are available from the corresponding author upon request.

## References

[1] Yachao Z (2017). Discussion on the solution strategy of coal-power contradiction in the context of power reform. Enterp. Reform Manag..

[2] Zhijiang L, Guanghu P, Shengliang L, Weihai F (2019). An analysis of coal power integration development strategy. Enterp. Manag..

[3] Bin W (2021). Strengthening the management of purchased coal to improve the operation guarantee ability of coal power and chemical integration. China Coal Indus..

[4] Zhiyuan G, Meihua Z, Hongjun P (2018). A study on the mechanism of coal and electricity supply tension and long-term contracts under price regulation. China Min..

[5] Linmei Y (2018). A study on coal power contradiction based on SCP paradigm. Mod. Econ. Inform..

[6] Shukui Yu (2019). A brief discussion on the problems and countermeasures of coal power integration. China Manag. Inform..

[7] Rui N, Zhenpeng T, Ruyi S (2017). Design of electricity and coal price formation mechanism from the perspective of bilateral matching. Price Theor. Pract..

[8] Daqing Z (2018). Analysis of the relationship between the short-term power of coal power manufacturers and coal power integration in China. J. Beijing Jiaotong Univ..

[9] Hongji S (2019). Research on the Vertical Relationship of China’s Coal Power Industry Under the New Normal.

[10] Yujia W (2019). Energy industry chain integration and enterprise productivity—Taking coal and electricity vertical integration as an example. J. Beijing Univ. Technol..

[11] Brown D P , Sappington D . Vertical Integration and Capacity Investment in the Electricity Sector. Working Papers, 2020.

[12] Guo H, Chen Q, Zhang Y (2020). Constraining the oligopoly manipulation in electricity market: A vertical integration perspective. Energy.

[13] Li H, Lu Y, Tao Z (2017). Vertical integration and firm productivity. J. Econ. Manag. Strateg..

[14] Pinopoulos IN (2019). On the welfare effects of vertical integration: Opportunism vs. double marginalization. Econ. Lett..

[15] Jinfang Y (2019). Research on the Financial Risk of Vertical Integration of Coal Listed Companies.

[16] Liu H, Xu ZT, Li M (2021). Heterogeneous supplier performance evaluation of gas turbine development projects based on ANP-TOPSIS. Modern Manuf. Eng..

[17] Zhang YZ, Ye CM, Geng XL (2019). A risk-based supplier selection method based on hesitant fuzzy generalized Choquet integral. Indus. Eng. Manag..

[18] Weng X, Dang W, Tian X (2020). Evaluation of manned space station payload component suppliers based on Monte Carlo data envelopment analysis. Sci. Technol. Manag. Res..

[19] Luhua F (2019). Research on the evaluation and management of building material suppliers based on entropy weight TOPSIS model. J. Chongqing Univ. Technol..

[20] Wu HY, Wang J, Lai R (2019). Research on product configuration method integrating multi-criteria supplier evaluation. Mech. Des. Res..

[21] Gamiy Yu, Liashok Ya, Kostenko V, Zavialova O, Kostenko T, Kostyrka O (2019). Applying European approach to predict coal self-heating in Ukrainian mines. Min. Miner. Depos..

[22] Jingman Ma, Yueqiang Li (2021). The construction of evaluation index system for the selection of suppliers of paper enterprises. Light Indus. Sci. Technol..

[23] Sakhardande MJ, Gaonkar RSP (2022). On solving large data matrix problems in Fuzzy AHP. Expert Syst. Appl..

[24] Fan WT, He YH, Han X, Feng YC (2021). A new model to identify node importance in complex networks based on DEMATEL method. Sci. Rep..

[25] Wu B, Chen HH, Huang W (2021). Safety risk assessment of railroad gas tunnel construction based on fuzzy-entropy power theory. J. Saf. Environ..

[26] Kepeng H, Lidie W (2021). An open pit slope hazard assessment model based on improved FAHP-CRITIC method and cloud theory. J. Saf. Environ..

[27] Yuan Z, Jun H, Wentao D, Wenyuan W (2021). The evaluation method of port competitiveness by cargo category based on the combined assignment-TOPSIS method. Sci. Technol. Eng..

[28] Pamuar D, Ecer F, Cirovic G (2020). Application of improved best worst method (BWM) in real-world problems. Mathematics.

[29] Biswas S, Pamucar D (2020). Facility location selection for b-schools in indian context: A multi-criteria group decision based analysis. Axioms.

[30] Uluta A, Karaku CB (2021). Location selection for a textile manufacturing facility with GIS based on hybrid MCDM approach. Ind. Text..

[31] Bai C, Kusi-Sarpong S, Badri Ahmadi H (2019). Social sustainable supplier evaluation and selection: A group decision-support approach. Int. J. Prod. Res..

[32] Kaviani MA, Yazdi AK, Ocampo L (2019). An integrated grey-based multi-criteria decision-making approach for supplier evaluation and selection in the oil and gas industry. Kybernetes.

[33] Wang CN, Tsai HT, Ho TP (2020). Multi-criteria decision making (MCDM) model for supplier evaluation and selection for oil production projects in Vietnam. Processes.

[34] Mukhametzyanov I (2021). Specific character of objective methods for determining weights of criteria in MCDM problems: Entropy, CRITIC and SD. Decis. Mak..

[35] Zarbakhshnia N, Jaghdani TJ (2018). Sustainable supplier evaluation and selection with a novel two-stage DEA model in the presence of uncontrollable inputs and undesirable outputs: A plastic case study. Int. J. Adv. Manuf. Technol..

[36] Stojanović I, Puška A (2021). Logistics performances of gulf cooperation council’s countries in global supply chains. Decis. Mak..

[37] Alosta A, Elmansuri O, Badi I (2021). Resolving a location selection problem by means of an integrated AHP-RAFSI approach. Rep. Mech. Eng..

[38] Badi I, Muhammad LJ, Abubakar M (2022). Measuring sustainability performance indicators using FUCOM-MARCOS methods. Oper. Res. Eng. Sci..

[39] Liu Y, Eckert CM, Earl C (2020). A review of fuzzy AHP methods for decision-making with subjective judgements. Expert Syst. Appl..

[40] Akram M, Arshad M (2019). A novel trapezoidal bipolar fuzzy TOPSIS method for group decision-making. Group Decis. Negot..

[41] Zindani D, Maity SR, Bhowmik S (2020). Interval-valued intuitionistic fuzzy TODIM method based on Schweizer-Sklar power aggregation operators and their applications to group decision making. Soft. Comput..

[42] Bouhedja S, Boukhaled A, Bouhedja A, Benselhoub A (2020). Use of the TOPSIS technique to choose the best supplier of quarry natural aggregate. Min. Miner. Depos..

